# National Dental Association, Niagara Falls, August 1–4, 1899

**Published:** 1899-10

**Authors:** J. M. Walker


### PROCEEDINGS.

### National Dental Association, Niagara Falls, August 1-4, 1899.

Reported for the DENTAL REGISTER by Mrs. J. M. Walker.

The second annual session of the National Dental Association held its meetings in the ball-room of the International Hotel, Niagara Falls, beginning at 11 A. M. Tuesday, August 1st.

The meeting was called to order by the President, Dr. H. J. Burkhart, Batavia, N. Y., who, after an opening prayer by Rev. A. S. Bacon, of Niagara, proceeded to read his annual address, of which a brief abstract follows. After extending a hearty welcome to all present, Dr. Burkhart dwelt upon the various measures inaugurated at the last annual session—the representation of the Association at the International Dental Congress in Paris, 1900; the establishment and increase of the

Army Medical Museum and Library, Washington, and the gathering of material for a reliable history of the dental profession.

Dr. Burkhart recommended the amendment of the by-laws so as to admit any clean, honest, ethical dentist, regardless of membership in State societies, a change in the method of electing delegates, the present method being too cumbersome and the requirements too restrictive; there should be no hesitation about a radical departure, in order that instead of a mere handful, the membership roll may consist of several thousands, at least. Dr. Burkhart said that he favored the adoption of the rules to be reported by the committee appointed to revise the Constitution and By-Laws, especially in the creation of an Executive Council to hold daily meetings for the disposal of all routine business. He favored immediate measures to obtain the appointment of dentists in the army and navy, and the unification of State laws, and the enactment of a law making a license granted in one State "a passport for practice not only on this continent but all over the world." He spoke of the necessity of correcting infirmary abuses and of a high order of ethical conduct on the part of all connected with dental colleges. He also favored the exacting of a higher preliminary standard of matriculation.

The address was referred to a committee consisting of Drs. James McManus, Thomas P. Hinman and L. P. Bethel, whose report was discussed at a later session.

Dr. J. N. Crouse asked the privilege of the floor, and announced that news had just been received that the "International Tooth Crown Co." had won a suit against a New York dentist, and that with the approval of the Association he would telegraph for the attorney of the Dental Protective Association, who would give a full and clear exposition of the present status.

A meeting of the Dental Protective Association was, therefore, called for Friday, to which all members of the Association were invited to be present.

The Secretary read a communication conveying greetings from the American Dental Society of Europe, and announced the presence of Dr. Wm. Mitchell, of London, and Dr. L. C. Bryan, of Switzerland, as representatives of that society. On motion of Dr. Patterson the courtesies of the floor were extended to the gentlemen who briefly acknowledged the courtesy, expressing sympathy in all matters concerning the interests of the dental profession, and a feeling of responsibility in upholding the name and dignity of the common calling, with a promise of seconding all the efforts of the parent organization.

REPORTS OF COMMITTEES.

Dr. Thomas Fillebrown, Chairman of the Committee on Revision of the Constitution.

On motion of Dr. Hunt, the Constitution, as revised and amended, was ordered printed and distributed before discussion for rejection or adoption.

The discussion was also deferred till after the report of the Committee on the President's Address, which is so largely in harmony with the proposed revisions.

Dr. Charles McManus, Chairman of the Committee on History, read portions of their report which was accepted as a whole and the committee continued, the chairman of the committee being authorized to fill the vacancy caused by the death of the lamented Dr. R. Finley Hunt.

The Treasurer offered his report showing $1,247 collected in dues since the last annual meeting, and a cash balance on hand of $954.72.

Dr. M. F. Finley, Chairman, read the report from the Committee on Dentists in the Army and Navy, giving the history of the rejection of the bill by Congress. It was stated that the Surgeon-General was not adverse to the innovation, but that he was not sufficiently impressed with its importance to offer such inducements as would secure efficient services.

The report was accepted, referred to the Committee on the President's Address and the committee continued.

The division on Credentials of the Executive Committee offered a resolution rescinding the vote of censure passed upon the New Jersey State Society in the matter of endorsing a certain nostrum.

The resolution was adopted.

Adjourned to 7:30 P. M.

*Evening Session.*

### THE SPECIALIZATION OF OUR PRELIMINARY EDUCATION.

BY DR. R. H. HOFHEINZ, ROCHESTER, N. Y.

After reviewing briefly what education was supposed to mean in the middle ages, the forming of young knights or of young monks, the conceptions of Erasmus, of Sturm, of Comenius and others, and the illumination thrown upon the problem of education by the doctrine of evolution, the essayist dwelt upon the importance of natural aptitudes and the advantages of specialization. A one-sided scientific education may become as harmful to a profession based upon esthetic and artistic training as a one-sided technical training would be. This is recognized in all the dental schools but the preliminary. Has it been recognized in the training of the dental student? Are the three years of the dental college course sufficient to train unskilled hands that have had no previous chance for the development of usefulness and skill ?

Preliminary education is bestowed upon the mind, while the executive functions of the physical system are ignored. A student who has not developed some manual skill under favorable conditions, at the age of eighteen, does not possess the physical requisites for a good dental operator; the student who is not naturally endowed with a reasonable amount of technical ability will never become an expert dental operator. Hence the necessity of the introduction into all secondary schools of drawing and constructive work.

The educational value of manual training has been established beyond all contravention. It develops in the pupil powers of thought and expression, and calls out the executive powers, giving self-confidence in dealing with actual material.

Public education in art is needed; the art spirit should enter into every-day labor. The public, or primary, school should take account of the esthetic faculty, which belongs to the higher nature of the child. It is the testimony of the most eminent educators that manual training does not interfere with academic advancement. On the contrary, it gives added intelligence in comprehending what is brought to the notice of the pupil and a wider range of thought.

Preliminary education must adjust itself to the needs of the future, and nowhere is this more necessary than to the future dentist. His early training must have a more specific direction —not at the expense of science, but for its benefit. Hand-in-hand with the training of the mind must be that of the hand and eyes. The intellectual culture of constructive art is as great as mental discipline—a great moral discipline—an incentive to greater honesty, for honesty is but the moral expression of exactness.

DISCUSSION.

In the discussion of this paper Dr. James Truman expressed the opinion that the most impressive consideration suggested by the paper is that a young man who has attained the age of eighteen years, without the opportunity of acquiring manual dexterity, will rarely or never acquire the practical skill essential to the successful dental operator. He suggested for the consideration of those who hold that the dental student should take a full medical course after his preliminary high school and college training before entering upon the study of dentistry, the question, What would be the age of that young man on entering the dental college, and what would be the probabilities as to his attainment of manual dexterity at that age?

Dr. G. V. Black said that he wished to endorse the views of the essayist and those expressed by Dr. Truman on this point. He was most heartily in accord with the expression of the necessity for acquiring manual training in early school life; of learning to use the hands to accomplish something; to produce something; to be useful. With all our boasted advancement there is yet much to be gained in the future, especially in the direction of manual skill, the mind and hand working together for good; for that which shall endure.

Dr. George D. Sitherwood spoke of the well-known fact that there comes a time in the life of a man when he can not learn to write. It takes years to train the hands to form the letters mechanically. He spoke regretfully of the growing tendency to confine the study of dentistry to the dental college, with "the faculty" as preceptors. The graduate who has had only the opportunities offered during his college life will find himself deficient in practical skill when he undertakes his first piece of bridge-work, or his first crown, if "the faculty" has been his only preceptor. They must have preliminary manual training before entering the dental college, and this should be acquired in the office of a competent preceptor.

Dr. Sitherwood's views on this point were not sustained by subsequent speakers.

Dr. Truman W. Brophy said that Dr. Sitherwood apparently had in mind the conditions existing twenty years ago. Through the technic work now done in the colleges the young man can get as much benefit in three months as he could in a year in my dental office.

Dr. C. N. Johnson feared there was danger in the increasing tendency towards specialization, which tends toward narrow-mindedness. The results of the *preceptor system* depend largely upon the preceptor, but it is an actual fact that the first year in the dental college is spent by the student in *unlearning* what has been learned with a preceptor.

Dr. Garrett Newkirk spoke of the correctness of the principle that unless a man develops mechanical skill early in life he will not be successful in the manipulative work of operative dentistry. It was formerly the fashion to study under a preceptor, but now the colleges prefer to take a boy who has done no such preliminary work and start him at once in technic work. He said the advances made along this line have been a revelation to me.

Dr. B. Holly Smith agreed that the boy should start early in his life's work if he expected to be skilled in manhood. The mind and hand should be developed and trained together. The old preceptor system was all right when the preceptor himself was all right, but often the so-called student was relegated to the laboratory and given charge of the vulcanizer, with an occasional chance to collect bills; in such a case his chances were not very good for becoming a skilled operator.

### DENTAL HISTORY.

Dr. B. J. Cigrand read a paper urging the importance of making the study of dental history a part of the dental college curriculum. Evidence of the progress of modern dental science is found in the ever-increasing interest manifested in historic research. Lessons of wisdom for the future are to be drawn from the events of the past. To the dental student the history of the progress of dental art and science must be held with the deepest interest.

There are at present six medical colleges which include the study of medical history in their course of instruction. The Illinois School of Dentistry was the first dental school to create a chair in dental history. Others will doubtless soon fall into line.

### DISCUSSION.

In the discussion of this subject Dr. Charles McManus expressed his appreciation of the interest taken by Dr. Cigrand in the subject of dental history, and hoped that the present agitation of the subject may yield satisfactory results. The subject of dental history, if properly put before the profession, is capable of doing great good. The medical profession prides itself upon its past, and points to Hippocrates, Esculapius, etc. If we would put ourselves on the same plane we must do all we can, as individuals, as colleges, as State societies, as National societies, to aid in the gathering up of material for a correct history of our past. We have a history; it runs back three thousand years. It is a good idea to give it a place in the dental college curriculum. The time is ripe for all dental colleges to teach dental history and to establish chairs of history.

Dr. W. C. Barrett said that he was more than anxious to see a proper history of dentistry—one that shall not include the many foolish traditions that have been handed down from the past, but which have no foundation in fact. The true historian must be able to sift fact from fable. The truths of history only must be taught in our schools.

Dr. G. V. Black said that in the study of medical history students in their fourth year are referred to the old volumes in which alone the records are to be found, and quoted in their reading, but there is no one volume in which it is all written out. It is a study which offers many difficulties, but the reward is sure.

In closing the discussion, Dr. Cigrand said that if each individual would feel that he had a personal interest in the matter a great deal could be accomplished. The material for a history of dentistry is in existence, but very much of it has yet to be discovered and made available.

### DENTAL ARTICULATION AND OCCLUSION.

BY WM. ERNEST WALKER, PASS CHRISTIAN, MISS.

The teeth have two articulations—the dento-alveolar articulation, their relationship with their alveoli, and the inter-dental articulation, a gliding movement of the lower upon the upper teeth, the latter being an important matter for consideration in the arrangement of cusps in prosthesis, in orthodontia, in contour operations, and in peridental diseases. Occlusion is the mere coming together of the teeth, principally cusps to salci—the act of closing the teeth and lips and keeping them closed. ("Occlusion, a shutting up; a closing."—*Century Dict.*)

It has, however, been suggested or recommended that in order to avoid the double use of the term, the word articulation be used only in its strictly anatomical sense, as describing the relationship of the teeth to their alveoli; the term occlusion to be used in reference to the relationship between antagonizing teeth.*

The "relationship between antagonizing teeth," however, presents itself in several widely varying aspects, more different each from the others than are the two modes of articulation.

While confusion may possibly arise from the double use of the term articulation, it becomes "confusion worse confounded" when we attempt by the use of the term occlusion to express not only the position of the teeth when the mouth is closed and the teeth brought together with cusps interlocking on both sides

*See *Dental Cosmos*, December, 1896, pp. 1023 and 1036, for tects of words the discontinuance of which has been recommended, together with the terms advised for use as substitutes.

simultaneously, but also to the expression of all the different relative positions the teeth may assume in the lateral and protrusive excursions of the mandible, as in biting, or in grinding food on the right side or on the left, in the function of mastication.

The generality of writers, therefore, use the words articulation and occlusion with the specific meaning applied to them by Dr. W. G. A. Bonwell in his well-known treatise, "The Geometrical and Mechanical Laws Governing the Articulation of the Human Teeth."

In the latest authority on this subject—"The American Text-book of Prosthetic Dentistry,"—Dr. Molyneaux says (p. 346): "The lower jaw has only one position of complete occlusion, and that is when both condyles are resting in the glenoid fossæ and the mouth is closed."

The positions in articulation are various, and in occlusion the teeth have a much greater amount of surface contact than in any position of articulation.

That the exclusive use of the term occlusion, to express all the phases of relationship between the antagonizing surfaces of the teeth, has not proven satisfactory to the profession at large, was shown by the essayist in the continued use of the term articulation by the best writers, not only in dental periodical literature, but especially by the authors of the different chapters in "The American Text-book of Prosthetic Dentistry," from which quotations were given from the chapters by Drs. Essig, Burchard, Goddard, Molyneaux, Ambler Tees, W. W. Evans and A. H. Thompson.

The use of the word articulation in reference to the antagonizing surfaces of the teeth, by Dr. E. S. Talbot, in his latest work, "Interstitial Gingivity," was pointed out; also the continued use in papers and discussions before the Academy of Stomatology (Philadelphia), the Institute of Stomatology (New York), the American Academy of Dental Science (Boston), the Section of Stomatology of the American Medical Association, the New York Odontological Society, and numerous other scientific bodies.

As it is always undesirable to make radical departures from long-established usage unless any decided advantages are apparent, the words articulation and occlusion having been long used in a clearly-defined and specific sense by the most prominent men in our ranks, it would seem advisable to continue to use them in that sense until some erudite scholar shall supply us with new terms free from the objectionable double application and more than partially expressive, though it is to be desired that proper discrimination should be made by the few who have used them as synonyms.

Discussion of this paper was deferred until after the reading of the other papers offered by Section 2.

